# Medikamentöse Ausstattung arztbesetzter Rettungsmittel – ist eine präklinische Therapie nach aktuellen Leitlinien möglich?

**DOI:** 10.1007/s10049-022-01036-6

**Published:** 2022-05-13

**Authors:** Eike Carstens, Hendrik Eismann, Markus Flentje, Thomas Albers, Lion Sieg

**Affiliations:** Klinik für Anästhesiologie und Intensivmedizin, Medizinische Hochschule Hannover, Carl-Neuberg-Str. 1, 30625 Hannover, Deutschland

**Keywords:** Rettungsdienst, Notfallmedizin, Organisation und Administration, Medikamente, Emergency medical services, Prehospital emergency care, Organization and administration, Pharmaceutical preparations

## Abstract

**Hintergrund:**

Eine hohe Versorgungsqualität in der präklinischen Notfallmedizin zeichnet sich durch eine leitliniengerechte Therapie aus. Grundvoraussetzung für diese Therapie ist das Vorhalten der benötigten Medikamente entsprechend den gültigen Leitlinienempfehlungen. Ob dies flächendeckend gewährleistet wird, ist aktuell unklar. Ein einheitlicher Standard zur medikamentösen Ausstattung arztbesetzter Rettungsmittel in Deutschland existiert nicht. Ziel der vorliegenden Arbeit ist die Identifikation von wichtigen Diagnosen und der zu ihrer Therapie benötigten Medikamente. Ein Abgleich dieser Ergebnisse mit der realen Ausstattung arztbesetzter Rettungsmittel ermöglicht die Bewertung hinsichtlich leitliniengerechter Therapieoptionen.

**Material und Methoden:**

Nach einer strukturierten Leitlinienrecherche wurden Tracerdiagnosen definiert und ihnen relevante Medikamente zugeordnet. Hier wurde auch der Evidenz- und Empfehlungsgrad berücksichtigt. In einem zweiten Schritt wurden Ärztliche Leitungen Rettungsdienst zu der Ausstattung der von ihnen verantworteten Rettungsmittel befragt und die Ergebnisse mit den empfohlenen Medikamenten verglichen.

**Ergebnisse:**

Insgesamt wurden 156 verschiedene Medikamente identifiziert. Der Median der vorgehaltenen Medikamente beträgt 58 bei einer minimalen Vorhaltung eines Standorts von 35 Medikamenten und maximaler Vorhaltung mehrerer Standorte von 77 Medikamenten.

**Diskussion:**

In der vorliegenden Erhebung wurden die in Leitlinien empfohlenen Medikamente mit der realen Ausstattung von arztbesetzten Rettungsmitteln verglichen. Insgesamt zeigt sich, verglichen mit einer Studie aus dem Jahr 2011, eine verbesserte Strukturqualität. Die empfohlenen Medikamente werden zu einem hohen Maß prähospital vorgehalten. Die Daten dieser Erhebung können von Rettungsdienstbereichen in ganz Deutschland zur Beurteilung ihrer individuellen Strukturqualität genutzt werden.

**Graphic abstract:**

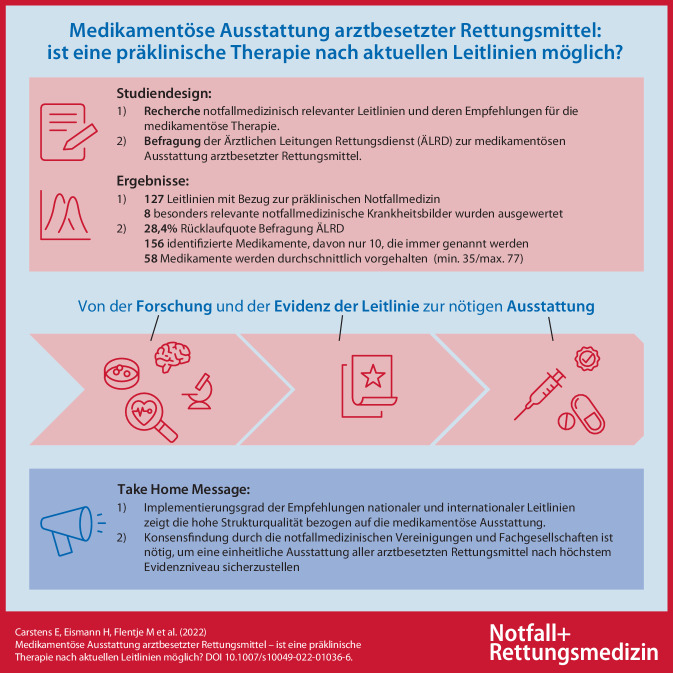

**Zusatzmaterial online:**

Die Online-Version dieses Beitrags (10.1007/s10049-022-01036-6) enthält ergänzende Übersichtstabellen.

## Abstract (en)

**Background:**

High quality of care in prehospital emergency medicine is characterized by guideline-based therapy. The basic prerequisite for this therapy is the availability of the required drugs in accordance with the current guideline recommendations. It is currently unclear whether this is guaranteed nationwide. There is no uniform standard regarding which drugs must be stocked in emergency medical services (EMS) vehicles staffed by physicians in Germany. The aim of the present study is to identify important diagnoses and the drugs required for their therapy. In a second step, medical directors throughout Germany were interviewed about current drugs available in their physician-staffed EMS vehicles and these were compared with the previously defined diagnosis-dependent drug lists.

**Materials and methods:**

After a structured guideline search, tracer diagnoses were defined and relevant drugs were assigned to them. The levels of evidence and recommendations were also considered. In a second step, this was compared with the current drugs available in physician-staffed EMS vehicles.

**Results:**

A total of 156 different medications were identified. The median number of medications stocked was 58; the minimum number of medications stocked was 35 at one site, while multiple sites stocked a maximum of 77 medications .

**Discussion:**

The present study investigated stocked medications in physician-staffed EMS vehicles. Overall, compared to a 2011 study, drug availability has improved. Most of the recommended medications are available in physician-staffed vehicles in Germany. The data from this study can be used by EMS throughout Germany to evaluate their preparedness.

Leitlinien wissenschaftlicher Fachgesellschaften sind Grundlage für eine optimale Patientenversorgung im Rahmen der evidenzbasierten Medizin. Leitlinien dienen hierbei dem Transfer von wissenschaftlicher Evidenz und Konsensempfehlungen von Fachexperten in den klinischen Alltag. Die Umsetzung dieser Leitlinien stellt einen wichtigen Qualitätsindikator der klinischen Versorgung dar [1]. Für die präklinische Versorgung von Notfallpatienten existieren Empfehlungen und Leitlinien unterschiedlichster Fachgesellschaften [2]. Die medikamentöse Ausstattung der arztbesetzten Rettungsmittel stellt eine Voraussetzung zur Umsetzung der Empfehlungen dar und kann somit als Implementierungsgrad angesehen werden. Die Ausstattung wird nach Empfehlung der Bundesärztekammer durch die Ärztliche Leitung Rettungsdienst (ÄLRD) des jeweiligen Rettungsdienstbereichs individuell vorgegeben [3]. Eine bundesweit einheitliche Ausstattung existiert nicht.

## Hintergrund und Fragestellung

Im Rahmen der prähospitalen Patientenversorgung in Deutschland konnten Rörtgen und Kollegen im Jahr 2011 zeigen, dass eine leitliniengerechte Therapie am Einsatzort nach höchstem Evidenzgrad aufgrund mangelnder medikamentöser Ausstattung häufig nicht erfolgen konnte [4]. Je nach definierter Tracerdiagnose war eine solche Therapie in 30–80 % der untersuchten Diagnosen nicht möglich [4]. Mit der vorliegenden Erhebung soll untersucht werden, ob sich in der vergangenen Dekade die medikamentöse Ausstattung der arztbesetzten Rettungsmittel den Leitlinienempfehlungen weiter angenähert hat. Der Implementierungsgrad der Empfehlung wird für notfallmedizinisch relevante Krankheitsbilder untersucht. Diese Tracerdiagnosen wurden aufgrund ihres gehäuften Auftretens in der präklinischen Notfallmedizin und der Notwendigkeit einer sofortigen Versorgung ausgewählt [2, 5]. Diese umfassen die anaphylaktische Reaktion, den schweren Asthmaanfall bzw. die akute Exazerbation einer chronisch-obstruktiven Lungenerkrankung (COPD), die kardiopulmonale Reanimation, die Schmalkomplextachykardie, den ST-Hebungs-Infarkt, die akute behandlungsbedürftige Hypo- oder Hypertonie, den Status eines generalisierten tonisch-klonischen Krampfanfalls sowie die prähospitale Notfallnarkose.

## Studiendesign und Untersuchungsmethoden

Die vorliegende Arbeit ist eine deskriptive Beobachtungsstudie, die in zwei Schritten erfolgte. Zunächst erfolgte eine Leitlinienrecherche hinsichtlich notfallmedizinisch relevanter Leitlinien und deren Empfehlungen für die medikamentöse Therapie. In einem zweiten Schritt führten wir eine Befragung der ÄLRD bezüglich der medikamentösen Ausstattung der arztbesetzten Rettungsmittel durch. Für die Studie lag ein positives Votum der Ethikkommission der Medizinischen Hochschule Hannover (Nr. 9191_BO_K_2020) vor.

### Leitlinienrecherche

Es erfolgte eine Schlagwortsuche innerhalb der Leitliniendatenbank der Arbeitsgemeinschaft der Wissenschaftlichen Medizinischen Fachgesellschaften e. V. (AWMF). Hierfür wurden die Schlagwörter „Notarzt“, „Notfallmedizin“, „prähospital“, „Präklinik“ sowie „Rettungsdienst“ verwendet. Die ermittelten Leitlinien wurden zunächst durch zwei Untersucher gesichtet und anhand des Titels wurde eine mögliche Relevanz für die Fragestellung erfasst. In einer zweiten Sichtung erfolgte durch fünf Untersucher mit mindestens 5‑jähriger Erfahrung in der Notfallmedizin die Identifizierung von präklinisch relevanten Empfehlungen. Für die oben genannten Tracerdiagnosen wurde ergänzend eine MEDLINE-Recherche durchgeführt und die nationalen Empfehlungen um die der europäischen Leitlinien ergänzt. Die Leitlinienempfehlungen werden, wenn möglich, für eine bessere Vergleichbarkeit hinsichtlich Empfehlungs- und Evidenzgrad nach dem Vorbild der European Society of Cardiology (ESC; [6]) dargestellt. Eine Erläuterung hierzu ist in den ergänzenden Übersichtstabellen im Online-Material dargestellt.

### Bestandsanalyse der vorgehaltenen Medikamente

Es erfolgte die Kontaktaufnahme über im Internet frei zugängliche Kontaktdaten von ÄLRD. Neben der Homepage des Bundesverbands der Ärztlichen Leiter Rettungsdienst e. V. (www.bv-aelrd.de) erfolgte eine Kontaktdatenakquise auch auf Bundeslandebene. Die ÄLRD wurden per E‑Mail kontaktiert und um Übersendung der Medikamentenausstattungslisten der arztbesetzten Rettungsmittel in ihrem Zuständigkeitsbereich gebeten. Alle ÄLRD, die nach drei Wochen keine Antwort übermittelt hatten, wurden maximal zweimal erneut angeschrieben.

### Datenauswertung und Statistik

Die Datenerhebung erfolgte pseudonymisiert. Die zugesendeten Medikamentenausstattungslisten wurden hinsichtlich der Wirkstoffe und Darreichungsformen ausgewertet. Die erhobenen Daten wurden mithilfe von Excel Version 14 (Microsoft, Redmond, WA, USA) deskriptiv erfasst. Kategoriale Variablen wurden als numerische Werte und prozentuale Anteile angezeigt.

## Ergebnisse

### Leitlinienrecherche

**Figure 1:**
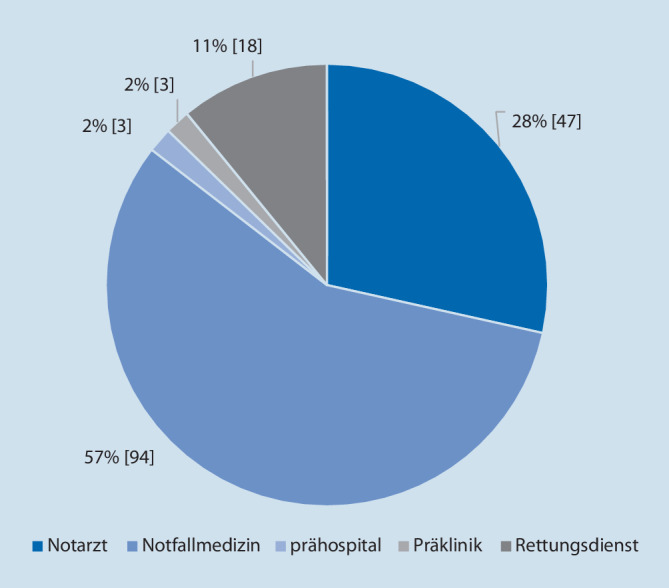

Am Erhebungstag konnten 127 Leitlinien identifiziert werden, die mindestens für eines der verwendeten Schlagwörter einen Treffer ergaben. Abb. 1 zeigt die Verteilung der Trefferhäufigkeit und der Gesamttreffer bei Möglichkeit der Mehrfachnennung.

**Figure 2:**
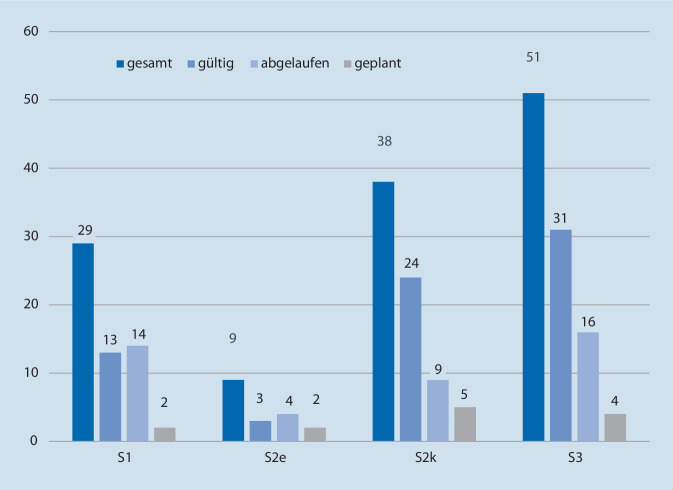

Nach der Stufenklassifikation des AWMF-Regelwerks [7] zur Planung und Organisation der Erstellung von Leitlinien entsprachen 51 Treffer der höchsten Stufe S3. Von diesen waren zum Untersuchungszeitpunkt 16 Leitlinien abgelaufen und weitere 4 lediglich angemeldet (Abb. 2).

Die Sichtung der 127 Leitlinien durch zwei Untersucher hinsichtlich einer möglichen Relevanz bezüglich der medikamentösen Ausstattung ergab für 27 Leitlinien die Nennung durch beide Untersucher. Hiervon waren 10 Leitlinien in ihrer Gültigkeit abgelaufen, wurden jedoch aufgrund ihrer vermuteten Relevanz im folgenden Schritt der Auswertung berücksichtigt. Darüber hinaus gab es weitere 16 Leitlinien (3 abgelaufene), die von jeweils einem Untersucher genannt wurden. Somit ergaben sich 43 Leitlinien, die durch die 5 erfahrenen Notfallmediziner begutachtet wurden. Die Sichtung der Leitlinien ergab, dass in 22 Leitlinien Empfehlungen unterschiedlicher Graduierung für eine medikamentöse Therapie in der Initialphase der Versorgung formuliert sind. Die MEDLINE-Recherche zeigte für 5 der 8 Tracerdiagnosen relevante Leitlinien von europäischen Fachgesellschaften.

### Befragung der ÄLRD

Für insgesamt 216 Rettungsdienstbereiche konnte ein ÄLRD identifiziert werden. Tab. 1 zeigt eine Übersicht nach Bundesländern. Hiervon haben 50 ÄLRD die angeforderten Listen zurückgeschickt, was einer Rücklaufquote von 22,7 % entspricht. Aufgrund der in Baden-Württemberg fehlenden landkreisbezogenen Strukturen hinsichtlich ÄLRD wurde Baden-Württemberg aus der Auswertung ausgeschlossen. Für das Bundesland Bayern existiert eine Empfehlung für eine einheitliche Vorhaltung von Notfallmedikamenten. Diese bayrische Bestückungsliste wurde als Einzelantwort in die Auswertung miteinbezogen. Unter Berücksichtigung dieser Sonderfälle ergibt sich somit für 176 befragte ÄLRD mit 50 Antworten eine Rücklaufquote von 28,4 %.

**Table 1:** 

Bundesland	Identifizierte ÄLRD	Antworten	Antwortquote [%]
Baden-Württemberg (BW)	4	0	0,0
Bayern	37	1^a^	2,7
Rheinland-Pfalz	7	2	28,6
Berlin	1	0	0,0
Brandenburg	17	4	23,5
Bremen	2	1	50,0
Hamburg	1	0	0,0
Hessen	20	4	20,0
Mecklenburg-Vorpommern	6	0	0,0
Niedersachsen	25	6	24,0
Nordrhein-Westfalen	53	20	37,7
Saarland	1	0	0,0
Sachsen	11	4	33,3
Sachsen-Anhalt	7	2	28,6
Schleswig-Holstein	10	2	18,2
Thüringen	14	4	28,6
*Gesamt*	216	50	23,1
*Gesamt exkl. BW und Einzelantwort Bayern*	176	50^a^	28,4

^a^Für das Bundesland Bayern existiert eine Empfehlung für eine einheitliche Vorhaltung von Notfallmedikamenten. Diese bayrische Bestückungsliste wurde als Einzelantwort in die Auswertung miteinbezogen

Insgesamt wurden 156 verschiedene Medikamente benannt. Der Median der vorgehaltenen Medikamente beträgt 58 bei einer minimalen Vorhaltung eines Standorts von 35 Medikamenten und maximaler Vorhaltung mehrerer Standorte von 77 Medikamenten. Alle genannten Wirkstoffe mit der zugehörigen Vorhaltung in Prozent sind bei absteigender Sortierung in den ergänzenden Übersichtstabellen im Online-Material dargestellt. In Tab. 2 sind die jeweiligen Medikamente anhand ihrer Tracerdiagnosen und der Vorhaltung in Prozent dargestellt.

**Table 2:** 

Empfehlungsgrad: Indikation	Nach Leitlinien geforderte Wirkstoffe und Applikationsweg	Vorhaltung (%)
**Anaphylaktische Reaktion **[11]		
I/A: wichtigste Substanz gegen alle Pathomechanismen der Anaphylaxie	Epinephrin intramuskulär (i.m.), ggf. intravenös (i.v.) oder inhalativ	100
I/A: stadienunabhängige Blockade der Histaminwirkung	Dimetinden i.v.	80
	Clemastin i.v.	16
I/C: supportiv bei schweren und therapieresistenten Anaphylaxien	Ranitidin i.v.	44
	Cimetidin i.v.	12
I/C: alle Formen der allergischen Reaktion	(Methyl‑)Prednisolon i.v.	100
I/A: Anaphylaxie mit führender bronchialer Obstruktion	Salbutamol inhalativ	94
	Reproterol i.v.	94
	Fenoterol inhalativ	26
	Terbutalin subkutan (s.c.)	22
**Asthmaanfall **[13]**/exazerbierte COPD **[14]		
I [13], I/B [14]: bei jeder Exazerbation schnellstmöglich	Salbutamol inhalativ	94
	Fenoterol inhalativ	26
I [13]: schwere Exazerbation	(Methyl‑)Prednisolon i.v.	100
I [13, 14]: bei unzureichender Wirkung von SABA	Ipratropiumbromid inhalativ	88
II [13]: bei unzureichender Wirkung der inhalativen Therapie	Reproterol i.v.	94
	Terbutalin s.c.	22
II [13]: schwere Exazerbation	Magnesiumsulfat i.v.	94
II [13]: nachgeordnet bei erhöhtem Nebenwirkungsrisiko III/B [14]: Verzicht wegen Nebenwirkungsrisiko	Theophyllin i.v.	22
Ausgleich einer metabolischen Azidose [13]	Natriumbikarbonat 8,4 % i.v.	66
Keine Erwähnung in den Leitlinien zur Akuttherapie [13, 14]	Beclometason inhalativ	14
	Budesonid inhalativ	8
**Kardiopulmonale Reanimation **[15]		
I: Alle Formen des Kreislaufstillstands	Epinephrin i.v.	100
I: Kammerflimmern, pulslose Ventrikuläre Tachykardie	Amiodaron i.v.	100
IIb: Alternativ zum Amiodaron	Lidocain i.v.	84
IIb: Hyperkaliämie, Trizyklikaintoxikationen	Natriumbikarbonat 8,4 % i.v.	66
IIa: Bei begründetem Verdacht auf Lungenarterienembolie	Fibrinolytika i.v. (Tenecte‑/Alte‑/Reteplase)	80
I: Bradykardie	Atropin i.v.	100
IIa: Bradykardie bei Beta‑/Kalziumkanalblockerintoxikation	Glukagon i.v.	4
I: Torsade-de-pointes-Tachykardie	Magnesiumsulfat i.v.	94
I: Bei Hyperkaliämie, Intoxikation mit Kalziumkanalblocker	Kalziumglukonat i.v.	48
**Schmalkomplextachykardie **[16]		
I/B: 1. Wahl bei Versagen vagaler Manöver	Adenosin i.v.	86
IIa/B: 2. Wahl bei Unwirksamkeit von Adenosin Einsatz eines Kalziumantagonisten	Verapamil i.v.	56
	Diltiazem i.v.	0
IIa/C: alternativ hierzu Einsatz Betarezeptorblocker	Metoprolol i.v.	100
**ST-Hebungs-Infarkt **[6]		
*Thrombozytenaggregationshemmung:*		
I/A: schnellstmögliche Gabe	Acetylsalicylsäure i.v.	100
I/A: Gabe eines ADP-Rezeptor-Antagonisten spätestens während der Katheterintervention	Acetylsalicylsäure per os (p.o.)	32
	Clopidogrel p.o.	6
	Ticagrelor p.o.	6
*Antikoagulationstherapie:*		
I/C: 1. Wahl als routinemäßige Gabe für alle ACS-Patienten	Heparin i.v.	98
	Enoxaparin i.v.	2
IIa/A: routinemäßiger Einsatz als Alternative zu unfraktioniertem Heparin sollte in Erwägung gezogen werden	Bivalirudin	0
IIa/A: Senkung des arteriellen Blutdrucks, wenn indiziert	Metoprolol i.v.	100
IIa/C: titrierte Gabe zur Schmerzlinderung	Morphin i.v.	100
I/C: Einsatz bei symptomatischer Herzinsuffizienz, wenn syst. RR > 90 mm Hg	Glyceroltrinitrat-Spray	98
IIa/C: Senkung des arteriellen Blutdrucks, wenn indiziert	Glyceroltrinitrat i.v.	26
**Hypertonie **[21]		
1. Wahl	Urapidil i.v.	96
1. Wahl bei pektanginösen Beschwerden, insbesondere bei akutem Koronarsyndrom und/oder Lungenödem zusätzlich	Glyceroltrinitrat i.v.	26
	Glyceroltrinitrat-Spray	98
	Furosemid i.v.	100
2. Wahl bei therapieresistenten hypertensiven Notfällen	Esmolol i.v.	0
	Alternativ: Metoprolol i.v.	100
	Enalaprilat i.v.	0
3. Wahl bei sehr schweren Fällen	Nitroprussid-Natrium i.v.	0
1. Wahl bei bestehender Agitiertheit, Unruhe oder Alkoholentzugssyndrom	Clonidin i.v.	30
Nicht eingesetzt werden sollten unretardierte Kalziumkanalblocker	Nitrendipin sublingual (s. l.)	44
	Nifedipin s. l.	2
**Hypotonie **[24, 25]		
*Katecholamine*		
IIa/A [24], I/B [25]: 1. Wahl Vasopressor	Noradrenalin i.v.	90
IIa/C [24], IIb/C [25]: 1. Wahl Inotropika	Dobutamin i.v.	50
IIb/C [24]: 2. Wahl	Epinephrin i.v.	100
Keine Empfehlung bei fehlender Evidenz [24]/Ergänzung zu Noradrenalin bei nicht ausreichender Einzelwirkung [25]	Vasopressin i.v.	2
Keine Erwähnung in den Leitlinien	Cafedrin/Theodrenalin i.v.	94
	Ephedrin i.v.	4
**Status generalisierter tonisch-klonischer Krampfanfall** [27]		
1. Wahl	Lorazepam i.v.	32
2. Wahl	Diazepam i.v.	38
	Clonazepam i.v.	24
Bei nicht verfügbarem i.v.-Zugang	Midazolam bukkal/nasal	22/96
	Diazepam rektal	72
Bei Nichtansprechen auf Benzodiazepine sollen i.v. applizierbare Antiepileptika eingesetzt werden	Phenytoin i.v.	12
	Valproat i.v.	0
	Levetiracetam i.v.	12
	Phenobarbital i.v.	4
Bei Nichtansprechen auf Antiepileptika	Thiopental i.v.	46
	Midazolam i.v.	96
	Propofol i.v.	88
**Prähospitale Notfallnarkose** [29–31]		
Hypnotika	Esketamin i.v.	100
	Midazolam i.v.	96
	Propofol i.v.	88
	Thiopental i.v.	46
Verzicht nahegelegt [29] bzw. empfohlen [30]	Etomidat i.v.	44
Muskelrelaxanzien	Rocuronium i.v.	90
	Succinylcholin i.v.	78
	Atracurium i.v.	2
	Cisatracurium i.v.	2
	Vecuronium i.v.	2
Opioide	Fentanyl i.v.	84
	Sufentanil i.v.	16

*i.m.* intramuskulär, *i.v.* intravenös, *s.c.* subkutan, *p.o.* per os, *s.l.* sublingual

## Diskussion

In der vorliegenden Studie wurde die medikamentöse Ausstattung arztbesetzter Rettungsmittel untersucht und mit den Empfehlungen von nationalen und europäischen Leitlinien verglichen.

Nach Empfehlung der Bundesärztekammer [3] unterliegt die pharmakologische Ausstattung der Festlegungshoheit der ÄLRD. Bereits in mehreren Publikationen [4, 8, 9] wurde eine Vereinheitlichung der Ausstattung, idealerweise auf nationaler Ebene, gefordert. Dies ist bis heute nicht erkennbar, wie die zum Teil noch immer relevanten Abweichungen von den Empfehlungen der Leitlinien zeigen. Das Bundesland Bayern hat hier mit einer zentral durch den Rettungsdienstausschuss Bayern verfassten Empfehlung zur einheitlichen Vorhaltung Vorbildcharakter [10].

### Therapie der anaphylaktischen Reaktion

In der zum Erhebungszeitpunkt aktuellen, jedoch bereits 2018 abgelaufenen Leitlinie zur Therapie der anaphylaktischen Reaktion von 2014 [11] wird Epinephrin (Vorhaltung 100 %) als das zentrale Medikament genannt. Trotz zum Teil geringer Evidenz [12] werden sowohl H1- als auch H2-Blocker empfohlen. Der Einsatz von H1-Blockern soll stadienunabhängig erfolgen und bei Therapieresistenz um einen H2-Blocker ergänzt werden. Die Vorhaltung ist zumindest für H1-Blocker mit 96 % gut, jedoch für H2-Blocker (66 %) erweiterbar. In der Leitlinie wird die untergeordnete Rolle der Glukokortikoide in der Akuttherapie wegen ihres verzögerten Wirkeintritts betont, dennoch sollen sie zur Prävention eines protrahierten oder biphasischen Verlaufs eingesetzt werden. β_2_-Sympathikomimetika werden bei führender bronchialer Obstruktion zusätzlich zum Epinephrin empfohlen. Mindestens ein Vertreter dieser Substanzgruppe ist auf allen Medikamentenlisten der Befragten zu finden. Insgesamt zeigt sich somit eine hohe Implementierung der Empfehlungen.

### Therapie eines schweren Asthmaanfalls bzw. einer akuten Exazerbation einer COPD

Die in den Leitlinien [13, 14] zum Teil bereits seit 2006 geforderten Medikamente zur Initialtherapie sind inzwischen in nahezu allen befragten Bereichen vorhanden. Im Vergleich zu 2011 hat die Verfügbarkeit von Salbutamol (41,1 % → 94 %), als häufigstem Vertreter der kurz wirksamen β_2_-Sympathomimetika (SABA), deutlich zugenommen [4]. Ebenso ist die Vorhaltung von Ipratropiumbromid (13,7 % → 88 %), welches nach aktuellen Empfehlungen bei unzureichender Wirkung der SABA einzusetzen ist, inzwischen nahezu flächendeckend. Somit wurde eine der zentralen Erkenntnisse der Arbeit von Rörtgen et al., die unzureichende Vorhaltung der empfohlenen antiobstruktiven Medikamente, nicht reproduziert. Zusätzlich werden in Ausnahmen indizierte Medikamente, wie das Theophyllin oder inhalative Kortikoide, weiterhin mitgeführt, die aus Sicht der Autoren keinerlei Stellenwert in der Akuttherapie haben. Natriumbikarbonat (66 %) zum Ausgleich einer metabolischen Azidose ist nach wie vor nicht flächendeckend verfügbar. Magnesiumsulfat zur Therapie bei initialem schlechtem Ansprechen und lebensbedrohlichen Anfällen wird hingegen mit 96 % vorgehalten.

### Kardiopulmonale Reanimation

Die zum Befragungszeitpunkt gültige Leitlinie des European Resuscitation Council (ERC; [15]) empfiehlt als zentrale Medikamente Epinephrin und Amiodaron, welche aktuell auf allen Rettungsmitteln vorgehalten werden. Neben diesen Substanzen werden weitere Medikamente für reversible Ursachen eines Kreislaufstillstands in der Leitlinie genannt. Bei begründetem Verdacht auf eine Lungenarterienembolie kann eine fibrinolytische Therapie erwogen werden. Eine Vorhaltung der hierfür möglichen Medikamente ist auf 80 % der Rettungsmittel gegeben. Hier zeigt sich eine Zunahme der Vorhaltung gegenüber 66,3 % in 2011 [4].

In dem Kapitel zu Peri-Arrest-Arrhythmien werden unverändert seit 2010 weitere Medikamente empfohlen. Für Bradykardien wird Atropin als Mittel der ersten Wahl empfohlen und flächendeckend vorgehalten. Bei einer ursächlichen Bradykardie durch Intoxikation mit Beta- oder Kalziumkanalblockern ist die Wirkung von Atropin häufig nicht ausreichend. Hier empfiehlt die Leitlinie den Einsatz von Glukagon. Die Vorhaltung dieser Substanz ist mit 4 % sehr selten und sollte verbessert werden. Bei Breitkomplextachykardien wird neben Amiodaron der Einsatz von Magnesium bei der Sonderform der Torsade-de-pointes-Tachykardie empfohlen. Hier zeigt sich eine verbesserte Implementierung der Empfehlung mit 94 % gegenüber 2011 mit 45,3 % [4]. Für eine weitere empfohlene Substanz ist dieser positive Trend nicht erkennbar; die Vorhaltung von Kalziumglukonat ist leicht rückläufig (50,5 % → 48 %). Der Einsatz wird z. B. bei Hyperkaliämien zur Membranstabilisierung sowie bei der Intoxikation mit einem Kalziumkanalblocker empfohlen.

### Therapie Schmalkomplextachykardie

Die ESC veröffentlichte 2019 eine europäische Leitlinie zur Therapie von supraventrikulären Tachykardien [16]. Bemerkenswert ist, dass Adenosin trotz des hohen Empfehlungsgrads zur Therapie der Schmalkomplextachykardie noch immer nicht in allen Rettungsdienstbereichen vorhanden ist. Hier hat es zwar eine deutliche Steigerung der Vorhaltung in der vergangenen Dekade von 59 % auf 86 % gegeben, diese scheint aber auf Kosten der 2.-Wahl-Substanz Verapamil (92 % 2011 vs. 56 % 2021) erfolgt zu sein. Die Vorhaltung mit einem Betarezeptorblocker ist vermutlich aufgrund seines breiteren Einsatzspektrums flächendeckend gegeben. Insgesamt ist ein höherer Implementierungsgrad der Empfehlungen bezüglich der ersten Wahl gegenüber den Daten aus 2011 [4] zu sehen.

### Therapie des ST-Hebungs-Infarkts

Der ST-Hebungs-Infarkt stellt eine der häufigsten Notarztindikationen dar [17]. Die Empfehlungen der ESC/ERC-Leitlinien [6, 18], welche zum Erhebungszeitpunkt gültig waren, sind grundsätzlich flächendeckend in der deutschen präklinischen Versorgung umsetzbar. Im Bereich der Thrombozytenaggregationshemmung scheint die Initialdosis von ADP-Rezeptor-Antagonisten präklinisch keine Anwendung zu finden. Sichtbar wird dieser präklinische Verzicht in der rückläufigen Vorhaltung von Clopidogrel von 30,5 % (2011 [4]) auf aktuell 6 %.

Die Empfehlung zum Einsatz von Morphin zur notwendigen Analgesie ist aufgrund einer möglichen Interaktion mit ADP-Rezeptor-Antagonisten im Vergleich zu früheren Versionen herabgestuft worden. Eine 2019 veröffentlichte Metaanalyse [19] hält den Einsatz jedoch weiter, trotz nicht zufriedenstellender wissenschaftlicher Datenlage, für gerechtfertigt.

### Therapie der akut behandlungsbedürftigen Hypo- oder Hypertonie

Abweichungen des Blutdrucks von den Normwerten sind ein häufiges, oft auch akut behandlungsbedürftiges Symptom im Rettungsdienst. Für die Senkung hypertensiver Werte stehen verschiedene Substanzen zur Verfügung. Die gemeinsame europäische Leitlinie „*Guidelines for the management of arterial hypertension*“ der ESC und der European Society of Hypertension (ESH) aus dem Jahre 2018 ist hier richtungsweisend [20]. In der deutschen Version [21] und Kommentierung [22] wird in dem neuen Kapitel zur Therapie des hypertensiven Notfalls auf zulassungsbedingte deutsche Besonderheiten eingegangen. Die international als Mittel der 1. Wahl empfohlenen Substanzen Labetalol und Nicardipin verfügen in Deutschland über keine Zulassung. Alternativ wird die Verwendung von Urapidil für die meisten Indikationen empfohlen. Hier zeigt sich auch eine nahezu flächendeckende Vorhaltung der Substanz von 96 %. Für die Behandlung der Hypertonie im Rahmen eines akuten Koronarsyndroms und/oder Lungenödems wird die Therapie mit intravenösem Nitroglycerin als direkter Vasodilatator, gegebenenfalls erweitert um ein Schleifendiuretikum, empfohlen. Der intravenöse Einsatz von Nitroglycerin mit dem Ziel der Blutdrucksenkung bei gleichzeitiger Verbesserung der Koronarperfusion scheint jedoch in der deutschen präklinischen Notfallversorgung nur eine untergeordnete Rolle zu spielen. Lediglich auf 26 % der Medikamentenlisten wird die Substanz geführt. Hier wird der sublingualen Applikation als Spray der Vorzug gegeben, was eine Vorhaltung von 98 % zeigt.

Der aufgrund seiner kurzen Halbwertszeit und daraus resultierenden guten Steuerbarkeit besonders empfohlene Betarezeptorblocker Esmolol wurde bei keiner der Antworten genannt. Das alternativ vorgeschlagene Metoprolol hingegen wird auf jeder Liste geführt. Weder in der englischsprachigen Originalpublikation noch in der deutschen Übersetzung wird für die Substanzgruppe der Betarezeptorblocker eine Differenzierung nach Empfehlungsgrad vorgenommen. Insgesamt kann für die präklinische Therapie bei zu hohen Blutdruckwerten ein sehr hoher Implementierungsgrad der Leitlinienempfehlungen der 1. und 2. Wahl festgestellt werden. Lediglich für das bei bestehender Agitiertheit, Unruhe oder Alkoholentzugssyndrom empfohlene zentrale Sympatholytikum Clonidin trifft dies mit 30 % nicht zu. Aufgrund der deutlichen Verzichtsempfehlung für den Einsatz von sublingual verabreichten, unretardierten Kalziumkanalblockern wie Nitrendipin (44 %) und Nifedipin (2 %) ist hier eine Anpassung der Medikamentenbestückung sinnvoll.

Die medikamentöse Therapie einer kritischen Hypotension ist zum einen bei der Versorgung von Patienten mit schwerem isoliertem Schädel-Hirn-Trauma [23] indiziert, zum anderen bei Patienten im Schock jedweder Genese. Im Schock sollte initial ein Volumenmangel ausgeschlossen oder gegebenenfalls korrigiert werden. Die medikamentöse Therapie der Hypotension erfolgt mittels Vasopressoren. Detaillierte Empfehlungen liefern die S3-Leitlinien zur Therapie des kardiogenen [24] und des septischen [25] Schocks. Die Vorhaltung von Noradrenalin als Vasopressor der 1. Wahl hat sich gegenüber der Erhebung 2011 [4] deutlich gesteigert (65,3 % → 90 %). Anders ist die Entwicklung bei dem Inotropikum der 1. Wahl Dobutamin. Hier zeigt sich ein Rückgang von 57,9 % auf 50 %. Das in früheren Leitlinien noch empfohlene, inzwischen jedoch obsolete Dopamin ist aus den Bestückungslisten gänzlich verschwunden. Vermutlich aufgrund der für die Akutmedizin einfacheren Handhabung als titrierbare Substanz wird Cafedrin/Theodrenalin häufig vorgehalten [26]. Eine Empfehlung in den Leitlinien ist für diese Substanz jedoch nicht zu finden.

### Therapie des Status generalisierter tonisch-klonischer Krampfanfall

Auf Grundlage einer 2012 veröffentlichten Leitlinie [27] der Deutschen Gesellschaft für Neurologie (DGN) ist Lorazepam das Antikonvulsivum der 1. Wahl in der Initialphase zur Beendigung eines generalisierten Krampfanfalls. Die Empfehlung erfolgt ohne Angaben über Empfehlungsstärke oder Evidenzgrad. Lediglich 32 % der ausgewerteten Medikamentenlisten führen intravenös zu verabreichendes Lorazepam auf. Verglichen mit den Daten von Rörtgen et al. [4] zeigt sich hier zwar eine deutliche Steigerung (13,7 % → 32 %), jedoch noch immer keine zufriedenstellende Implementierung der Empfehlung. Ursächlich für die nach wie vor geringe Vorhaltung könnte die Notwendigkeit der gekühlten Lagerung sein. Die zum Befragungszeitpunkt letztveröffentlichte Leitlinie der DGN ([27]; abgelaufen 09/2017) empfiehlt als intravenöses Mittel der 2. Wahl Diazepam oder Clonazepam. Hier hat sich die Vorhaltung gegenüber 2011 bei Diazepam von 84 % auf 38 % deutlich verschlechtert. Die Leitlinie sieht bei Nichtetablierung eines i.v.-Zugangs die rektale Gabe von Diazepam (Vorhaltung 72 %) bzw. bukkale (Buccolam-Fertigspritzen 22 %) oder intranasale Gabe von Midazolam vor. Die intranasale Gabe von zur i.v.-Gabe bestimmtem Midazolam ist weitverbreitet, stellt aber letztlich einen „off label use“ dar [28]. Midazolam wird von allen in der Leitlinie genannten Substanzen am häufigsten vorgehalten (96 %), ist jedoch nach der Leitlinie erst bei fortbestehender Therapieresistenz und Nichtansprechen auf zu bevorzugende Benzodiazepine und Antiepileptika intravenös einzusetzen. In der aktuellsten, nach der vorliegenden Befragung veröffentlichten Leitlinienversion sind alle Benzodiazepine gleichermaßen als Mittel der 1. Wahl definiert. Somit ist inzwischen die Vorhaltung als leitliniengerecht anzusehen, wies jedoch zum Erhebungszeitpunkt einen geringen Implementierungsgrad auf. Die Gruppe der Antiepileptika, laut Leitlinie Mittel der 2. Wahl bei Nichtansprechen auf Benzodiazepine noch vor der Gabe von Anästhetika, wird insgesamt nur gering vorgehalten (Levetiracetam und Phenytoin je 12 %).

### Prähospitale Notfallnarkose

Im Wesentlichen befassen sich zwei S1-Leitlinien der Deutschen Gesellschaft für Anästhesiologie und Intensivmedizin e. V. (DGAI) und die S3-Leitlinie Polytrauma/Schwerverletzten-Behandlung unter Federführung der Deutschen Gesellschaft für Unfallchirurgie e. V. mit der prähospitalen Notfallnarkose [29, 30] und dem dazugehörigen Atemwegsmanagement [31]. Es erfolgen keine konkreten Empfehlungen, welche Substanzen innerhalb der Gruppe der Hypnotika, Analgetika oder Muskelrelaxanzien zu bevorzugen sind. Vielmehr gibt die Leitlinie [29] zur prähospitalen Notfallnarkose der DGAI für verschiedene Szenarien unterschiedliche Handlungsempfehlungen. Für die Gruppe der Hypnotika kann allgemein [29] bzw. sollte bei traumatisierten Patienten [30] auf die Gabe von Etomidat zugunsten anderer Substanzen verzichtet werden. Begründet wird dies mit den Nebenwirkungen und möglicherweise relevanten Auswirkungen auf Morbidität und Letalität. Die Vorhaltung auf 44 % der Bestückungslisten bildet diese Verzichtsempfehlung nicht ab. Aus Sicht der Autoren erscheint das Vorhalten zweier unterschiedlicher Hypnotika sinnvoll, sodass die jeweiligen pharmakologischen Eigenschaften und die Patientencharakteristika berücksichtigt werden können. Hier zeigt sich deutlich eine stärkere Vorhaltung von Propofol (88 %) gegenüber Thiopental (46 %) und auch Etomidat (44 %). Diese Vorhaltung erscheint sinnvoll, da bei Propofol aufgrund der Verbreitung als Standardhypnotikum in der deutschen Kliniklandschaft von den höchsten Kenntnissen im Umgang mit der Substanz ausgegangen werden kann. Ergänzend zu Propofol sollte Esketamin (100 %), vor allem für traumatologische Patienten, vorgehalten werden. Alle genannten Leitlinien, die sich mit der prähospitalen Narkose befassen, empfehlen mit hohem Evidenzgrad und hoher Empfehlungsstärke die Vorhaltung eines Muskelrelaxans mit kurzer Anschlagszeit. Auf jeder zugesandten Liste findet sich mindestens eine der möglichen Substanzen Rocuronium (90 %) oder Succinylcholin (78 %). Gleiches gilt für die einsetzbaren Opioide. Fentanyl (84 %) wird deutlich häufiger genannt als Sufentanil (16 %). Grund hierfür könnte sein, dass für Sufentanil die Zulassung als reines Analgetikum ohne Intubationsnarkose fehlt [29]. Insgesamt zeigen die vorliegenden Bestückungslisten eine hervorragende Strukturqualität für die zur prähospitalen Narkose benötigten Medikamente.

## Limitationen

Die Befragung der ÄLRD erfolgt zu einem ungünstigen Zeitraum im Rahmen der SARS-CoV-2-Pandemie (August bis November 2020). Pandemiebedingt ist von einer hohen Arbeitsbelastung der ÄLRD auszugehen. Dies könnte ein Grund für die mit Rörtgen [4] verglichen geringere Rücklaufquote sein. Schwierigkeiten bereitet das Fehlen eines zentralen ÄLRD-Registers, welches Projektdurchführungen zum Thema prähospitales ärztliches Qualitätsmanagement erschwert. Für die vorliegende Arbeit bedeutet dies, dass die Antwortzahlen im Kontext einer Gesamtzahl nicht zu bewerten sind. Auch die unterschiedlichen länderbezogenen Strukturen erschweren diese Bewertung.

## Fazit

Insgesamt zeigt sich verglichen mit den Daten von Rörtgen et al. eine verbesserte Implementierung der Leitlinienempfehlungen. Rörtgen und Kollegen mussten 2011 noch feststellen, dass für bestimmte Tracerdiagnosen eine medikamentöse Therapie nach höchstem Evidenzgrad mit einer Häufigkeit von bis zu 80 % nicht möglich war [4]. Im Vergleich zu 2011 bestehen aktuell deutlich geringere Diskrepanzen gegenüber den Empfehlungen der Leitlinien. Insbesondere die Vorhaltung der Medikamente (Tab. 2) zur Akuttherapie der akuten Bronchokonstriktion und der Schmalkomplextachykardie und auch die Vorhaltung von Noradrenalin als Vasopressor der 1. Wahl haben sich verbessert. Der Implementierungsgrad der Empfehlungen der verschiedenen nationalen und internationalen Leitlinien ist insgesamt als hoch anzusehen und zeigt die hohe Strukturqualität bezogen auf die medikamentöse Ausstattung. Wir hoffen, dass die Datengrundlage dieser Arbeit von den jeweiligen Rettungsdienstbereichen zur Messung und möglichen Anpassung ihrer eigenen Strukturqualität genutzt wird.

Um tatsächlich eine einheitliche Ausstattung aller arztbesetzten Rettungsmittel nach höchstem Evidenzniveau sicherzustellen, wäre der bereits vor 20 Jahren formulierte Vorschlag zur Konsensfindung durch die Deutsche Interdisziplinäre Vereinigung für Intensiv- und Notfallmedizin e. V. (DIVI) und der Bundesvereinigung der Arbeitsgemeinschaften der Notärzte Deutschlands e. V. (BAND) sowie des Bundesverbands der Ärztlichen Leiter Rettungsdienst Deutschland e. V. sinnvoll.

## Supplementary Information



