# Supplementary material for: Stocked medications in emergency medical service vehicles staffed by physicians—is prehospital treatment according to current guidelines possible?
Source: Notf Rett Med. 2022 May 13:1–10. [Article in German] Online ahead of print. doi: 10.1007/s10049-022-01036-6 (PMC9101990; doi:10.1007/s10049-022-01036-6)
Supplement: Supplementary file 1 [file 10049_2022_1036_MOESM1_ESM.pdf]

Zusatzmaterial zum Beitrag „Medikamentöse Ausstattung arztbesetzter Rettungsmittel – ist eine präklinische Therapie nach aktuellen Leitlinien möglich?" von Carstens E, Eismann H, Flentje M et al. (2022) in *Notfall+Rettungsmedizin*

Beitrag und Zusatzmaterial stehen Ihnen auf www.springermedizin.de zur Verfügung. Bitte geben Sie dort den Beitragstitel in die Suche ein.

Empfehlungs- und Evidenzgrade nach European Society of Cardiology (ESC)

Empfehlungs-grad	Definition	Empfohlene Formulierung
**I**	Evidenz und/oder allgemeine Übereinkunft, dass eine Therapieform oder eine diagnostische Maßnahme effektiv, nützlich oder heilsam ist	Wird empfohlen / ist indiziert
**II**	Widersprüchliche Evidenz und/ oder unterschiedliche Meinungen über den Nutzen/die Effektivität einer Therapieform oder einer diagnostischen Maßnahme	
**IIa**	Evidenzen/ Meinungen favorisieren den Nutzen bzw. die Effektivität einer Maßnahme	sollte erwogen werden
**IIb**	Nutzen/ Effektivität einer Maßnahme ist weniger gut durch Evidenzen/Meinungen belegt	kann erwogen werden
**III**	Evidenz und/oder allgemeine Übereinkunft, dass eine Therapieform oder eine diagnostische Maßnahme nicht effektiv, nicht nützlich oder nicht heilsam ist und im Einzelfall schädlich sein kann	wird nicht empfohlen
**Evidenzgrad**		
**A**	Daten aus mehreren, randomisierten klinischen Studien oder Meta-Analysen	
**B**	Daten aus einer randomisierten klinischen Studie oder mehreren großen nicht randomisierten Studien	
**C**	Konsensusmeinung von Experten und/oder kleinen Studien, retrospektiven Studien oder Registern	

Vorgehaltene Medikamente auf ärztlich besetzten Rettungsmitteln,

Vorhaltung in %, per os (p.o.), intramuskulär (i.m.), intravenös (i.v.), subkutan (s.c.).

Wirkstoff	Darreichung	%
Acetylsalicylsäure	i.v.	100
Amiodaron	i.v.	100
Atropinsulfat	i.v.	100
Epinephrin	i.v.	100
Esketamin	i.v.	100
Furosemid	i.v.	100
Metoprolol	i.v.	100
Morphin	i.v.	100
(Methyl-)Prednisolon	i.v.	100
Vollelektrolyt-Lösung	i.v.	100
Glyceroltrinitrat	Spray	98
Heparin	i.v.	98
Naloxon	i.v.	98
Butylscopolaminiumbromid	i.v.	96
Midazolam	i.v.	96
Prednison	Zäpfchen	96

Urapidil	i.v.	96
Atropinsulfat 100mg	i.v.	94
Cafedrin / Theoadrenalin	i.v.	94
Flumazenil	i.v.	94
Magnesiumsulfat	i.v.	94
Reproterol	i.v.	94
Salbutamol	inhalativ	94
Tranexamsäure	i.v.	92
NaCl 0,9 % 10 ml	i.v.	90
Noradrenalin	i.v.	90
Rocuronium	i.v.	90
Dimenhydrinat	i.v.	88
Ipratropiumbromid	inhalativ	88
Metamizol-Natrium	i.v.	88
Propofol 1 %	i.v.	88
Adenosin	i.v.	86
Fentanyl	i.v.	84
Lidocain	i.v.	84
Aktivkohle	p.o.	80
Dimetinden	i.v.	80
Paracetamol	Zäpfchen	80
Succinylcholin	i.v.	78
Diazepam	rectal	72
Oxytocin	i.v.	72
Glucose 40%	i.v.	70
Lorazepam	p.o.	68
Dimethylaminophenol (4DMAP)	i.v.	66
Natriumbikarbonat 8,4%	i.v.	66
Toluidinblau	i.v.	66
Fenoterol	i.v.	64
NaCl 0,9 % 100 ml	i.v.	64
Aqua dest	i.v.	62
Haloperidol	i.v.	62
Tenecteplase	i.v.	62
Piritramid	i.v.	60
Verapamil	i.v.	56
Dobutamin	i.v.	50
Ondansetron	i.v.	50
Physostigmin	i.v.	50
Calziumgluconat	i.v.	48
Biperiden	i.v.	48
Ajmalin	i.v.	46
Thiopental	i.v.	46
Etomidat	i.v.	44
Natriumthiosulfat	i.v.	44
Nitrendipin	sublingual	44
Ranitidin	i.v.	44
Hydroxocobalamin	i.v.	40
Diazepam	i.v.	38
Dimeticon/Simeticon	p.o.	34
Acetylsalicylsäure	p.o.	32
Glucose 5%	i.v.	32

Lorazepam	i.v.	32
Paracetamol	i.v.	32
Clonidin	i.v.	30
Ibuprofen	Zäpfchen	30
Glyceroltrinitrat	i.v.	26
Fenoterol	inhalativ	26
Clonazepam	i.v.	24
Glucose 10%	i.v.	22
Obidoximchlorid	i.v.	22
Terbutalin	s.c.	22
Theophyllin	i.v.	22
Midazolam	p.o.	22
Promethazin	i.v.	20
Ceftriaxon	i.v.	18
Gelafundin	i.v.	18
Ibuprofen	Saft	18
Alteplase	i.v.	16
Glucose 20%	i.v.	16
Metoclopramid	i.v.	16
Oxybuprocain	Augentropfen	16
Sufentanil	i.v.	16
Clemastin	i.v.	16
Beclometasondipropionat	inhalativ	14
Granisetron	i.v.	14
Cimetidin	i.v.	12
Hydroxyethylstärke	i.v.	12
Levetiracetam	i.v.	12
NaCl 0,9 % 500 ml	i.v.	12
Nalbuphin	i.v.	12
Phenytoin	i.v.	12
Cefotaxim	i.v.	10
Dexamethason	i.v.	10
Epinephrin - InfectoKrupp	inhalativ	10
Mepivacain 1 -2%	peri neural	10
Budesonid	inhalativ	8
Dobutamin	Zäpfchen	8
Ondansetron	i.v.	8
Physostigmin	p.o.	8
Chitosan	Hämostyptikum	6
Clopidogrel	p.o.	6
NaCl 0,9 % 250 ml	i.v.	6
Orciprenalin	i.v.	6
Phosphatpuffer	Augenspülung	6
Reteplase	i.v.	6
Sugammadex	i.v.	6
Terlipressin	i.v.	6
Ticagrelor	p.o.	6
Ammoniak Lavendel Riechstäbchen	nasal	4
Diazepam	p.o.	4
Diclofenac	p.o.	4
Ephedrin	i.v.	4
Glucagon	i.v.	4

Glucose 50%	i.v.	4
Glucose Gel	p.o.	4
Jubin (Glucose)	p.o.	4
Methoxyfluran	inhalativ	4
Naloxon	nasal	4
Penicillin	i.v.	4
Phenobarbital	i.v.	4
Proparacain	Augentropfen	4
Proximetacin	Augentropfen	4
Thiamin	i.v.	4
Xylometazolin 0,1%	nasal	4
Atracurium	i.v.	2
Benperidol	i.v.	2
Calziumglukonat Gel	topisch	2
Cisatracurium	i.v.	2
Dehydrobenzperidol	i.v.	2
Digitoxin	i.v.	2
Digoxin	i.v.	2
Diphoterin	Augenspülung	2
E 148 P1	i.v.	2
Enoxaparin	s.c./i.v.	2
Etilifrin	p.o.	2
Gelaspon-Schwamm	Hämostyptikum	2
Ibuprofen	p.o.	2
Insulin	s.c.	2
Kaliumchlorid	i.v.	2
Kaolin	Hämostyptikum	2
Lipovenös	i.v.	2
Methylergometrin	i.v.	2
Nifedipin	sublingual	2
Parafinöl	p.o.	2
Parasorb	Hämostyptikum	2
Piperacillin/Tazobactam	i.v.	2
Vasopressin	i.v.	2
Vecuronium	i.v.	2
